# Prognostic Significance of CREB-Binding Protein and CD81 Expression in Primary High Grade Non-Muscle Invasive Bladder Cancer: Identification of Novel Biomarkers for Bladder Cancer Using Antibody Microarray

**DOI:** 10.1371/journal.pone.0125405

**Published:** 2015-04-27

**Authors:** Myung-Shin Lee, Joo Heon Kim, Ji-Su Lee, Seok Joong Yun, Wun-Jae Kim, Hanjong Ahn, Jinsung Park

**Affiliations:** 1 Department of Microbiology, Eulji University School of Medicine, Daejeon, South Korea; 2 Department of Pathology, Eulji University School of Medicine, Daejeon, South Korea; 3 Department of Urology, College of Medicine, Chungbuk National University, Cheongju, South Korea; 4 Department of Urology, Asan Medical Center, University of Ulsan College of Medicine, Seoul, South Korea; 5 Department of Urology, Eulji University Hospital, Eulji University School of Medicine, Daejeon, South Korea; Oklahoma University Health Sciences Center, UNITED STATES

## Abstract

High-grade (HG) bladder cancers (BCs) are genetically unstable and have an unpredictable course. The identification of prognostic factors in HG non-muscle invasive BC (NMIBC) is crucial for improving patients’ quality of life and preventing BC-specific mortality. Here, we used an antibody microarray (AbM) to identify novel candidate biomarkers in primary HG NMIBC and validated the prognostic significance of the candidate biomarkers. Three pairs of tissue samples from primary HG NMIBC and normal urothelium were analyzed using an AbM kit containing 656 antibodies, and differentially expressed proteins were identified. Among the 42 upregulated and 14 downregulated proteins with statistical significance in BC tissues, CREB-binding protein and CD81 were selected as representative upregulated and downregulated candidate biomarkers, respectively. We then validated the expression of these candidate biomarkers in primary human urothelial cells and BC cell lines by western blotting and immunofluorescence assays, and the results were consistent with the AbM expression profiles. Additionally, Kaplan-Meier survival using immunohistochemical data from an independent primary HG NMIBC cohort comprising 113 patients showed that expression of the 2 biomarkers was significantly associated with recurrence-free and progression-free survival. In multivariate analysis, the 2 biomarkers remained significant predictors for recurrence-free survival. Taken together, our findings suggest that expression of CREB-binding protein and CD81 in BC tissue specimens may have prognostic value in patients with primary HG NMIBC.

## Introduction

Bladder cancer (BC) is the second most common cancer of the genitourinary tract, and in 2008, an estimated 386,300 new cases of BC and 150,200 BC-related deaths occurred worldwide [1]. Urothelial carcinomas represent more than 90% of BC cases and are classified according to histologic grade as low-grade (LG) and high-grade (HG) tumors. It has been hypothesized that there are 2 distinct carcinogenesis pathways [2–4]. LG BC shows low recurrence and progression rates, whereas HG BC is frequently associated with tumor recurrence and progression. Despite complete removal of HG BCs by transurethral resection of bladder tumor (TURBT) with/without intravesical therapy, the recurrence rate is 40% to 78% within 5 years, and the progression rate is 4% to 45% [5, 6]. Thus, identification of prognostic factors in HG BC is essential to improve patients’ quality of life and prevent BC-specific mortality.

Compared to LG BCs, HG BCs are genetically unstable [7, 8]. The prognostic value of candidate molecular biomarkers in HG BCs has been inconsistent among studies due to tumor cell variations. In fact, we previously reported that several single biomarkers do not fully reflect the alterations in complex carcinogenesis pathways and the interrelationships between those pathways in HG BC [9]. Moreover, as the carcinogenesis and progression of HG BC are not a consequence of the action of a single protein, but rather of multiple proteins that function in pathways and networks, profiling BC-associated changes requires simultaneous measurement of many proteins in a single sample. Therefore, examination of multiple concurrent molecular alterations using a high-throughput technology could improve the identification of candidate prognostic molecular biomarkers [10]. While DNA microarrays have proved very useful for determining differential gene expression patterns in cancer, profiling the proteins expressed in cancer is much more challenging due to the complex regulation of protein expression and posttranslational modification. Recently, antibody microarrays (AbMs) have been developed to facilitate high-throughput proteomic studies. Although a few studies have analyzed BC using AbMs [11–14], an AbM has not yet been used to analyze the differences between BC and normal urothelial tissues.

In this study, we used an AbM containing 656 antibodies to identify novel biomarkers in primary HG non-muscle invasive BC (NMIBC). Among the proteins that showed distinctive expression in the AbM profiling, 2 representative proteins, CREB-binding protein (CREBBP) and CD81, were chosen, and their prognostic significance for tumor recurrence and progression were investigated in an independent primary HG NMIBC cohort. The 2 candidate biomarkers, CREBBP and CD81, examined in this study have not been well characterized in BC. This study is the first to demonstrate a significant correlation between the expression of CREBBP and CD81 and adverse pathological characteristics in NMIBC and their prognostic value.

## Materials and Methods

### Ethics statement and study protocol

The study protocol was approved by the Ethics Committee of Eulji University Hospital (Approval No. 11–0081) and adhered to the tenets of the Declaration of Helsinki. In addition, written informed consent was obtained from all study subjects. In the present study, we focused on urothelial carcinomas and excluded other BC histologic variants (squamous, micropapillary, sarcomatoid, small cell, and adenocarcinoma). Tumors were staged and graded according to the 7th American Joint Committee on Cancer criteria and the 2004 World Health Organization grading system [15]. The biomarkers reported in this study follow the REMARK guidelines [16]. All TURBT surgeries were performed with a curative intent using a standard technique as previously described [17].

### AbM profiling of human bladder tissues

For the AbM analysis, paired tissue samples of BC and normal urothelium were acquired from 3 patients who were diagnosed with primary HG NMIBC following complete TURBT. Exclusion criteria were LG BC, recurrent HG BC, an absence of proper muscle in the TURBT specimen, and the presence of other malignancies. Given the possible field effect [18] of normal-appearing bladder mucosa surrounding the BC, patients with concomitant carcinoma in situ were also excluded. After the tissue samples were obtained intraoperatively, they were immediately frozen in liquid nitrogen and stored at -70°C until protein extraction.

The AbM was prepared and analyzed using an AbM assay kit with 656 antibodies (Fullmoon Biosystems, Sunnyvale, CA) according to the manufacturer’s guidelines, as previously described [19]. Briefly, protein was extracted using lysis beads in a protein extraction buffer (Fullmoon Biosystems) containing 1% protease inhibitor cocktail (Sigma, St. Louis, MO) and 1% phosphatase inhibitor cocktail. After extraction, the protein solution was purified using the gel matrix column included in the AbM kit. The concentration of purified sample was then measured using the BCA Protein Assay Kit (Pierce, Rockford, IL) and a NanoPhotometer (Implen, UK). The protein sample was then labeled using a biotin/dimethylformamide solution. The AbM slide was treated with 30 mL of blocking solution in a petri dish and incubated on a shaker for 1 hour at room temperature. After blocking, the slide was rinsed with Milli-Q grade water. The labeled samples were then mixed with 6 mL of coupling solution and incubated with the blocked microarray slide in a coupling dish on a shaker for 2 hours at room temperature. After coupling, the slide was washed 6 times with 30 mL of washing solution. Then, 30 μL of 0.5 mg/mL Cy3-streptavidin (GE Healthcare, Piscataway, NJ) was mixed with 30 mL of detection buffer. The slides were scanned at 10-μm resolution with a GenePix 4000B scanner (Molecular Devices LLC, Sunnyvale, CA), and the scanned images were gridded and quantified using GenePix software. The numeric data were analyzed using Genowiz 4.0 (Ocimum Biosolutions, India). Global normalization was used for data analysis. A 2-class t-test or paired t-test was performed to compare each experiment-reference group combination. The averages of normalized ratios were calculated by dividing the average normalized BC sample intensity by the average normalized normal urothelium sample intensity. The benchmarks for upregulation and downregulation were 1.3-fold and 0.77-fold, respectively. After analysis, the data were annotated using protein information in the UniProt database. A hierarchical clustering algorithm was used to evaluate the association of protein profiles and the study individuals, with Pearson correlation as the distance metric, where distance = (1—Pearson correlation value)/2, and the average linkage method, as described previously [12]. The array data were deposited in the NCBI’s Gene Expression Omnibus and are accessible through GEO Series accession number GSE59440 (http://www.ncbi.nlm.nih.gov/geo/query/acc.cgi?acc=GSE59440).

### Cell culture

Primary human urothelial cells were purchased from ScienCell Research Laboratories (Carlsbad, CA) and cultured in Urothelial Cell Medium (ScienCell Research Laboratories) according to the manufacturer’s recommendations. BC cell lines (RT4, T24, and TCC-SUP) were obtained from the Korean Cell Line Bank (Seoul, South Korea) or American Type Culture Collection (ATCC, Manassas, VA) and were cultured at 37°C in Dulbecco’s Modified Eagle’s Medium (Welgene, South Korea) or Eagle’s minimal essential medium supplemented with 10% fetal bovine serum (Gibco BRL, Grand Island, NY).

### Western blotting and immunofluorescence assay (IFA)

Western blotting and IFA were performed as described previously [20]. Polyclonal rabbit anti-CREBBP (Bioss Inc., Woburn, MA), anti-CD81 (Thermo Scientific, Rockford, IL) and anti-β-tubulin (Sigma) antibodies were used as primary antibodies. For IFA, samples were examined using a Nikon Eclipse E400 microscope (Nikon Instruments Inc., Melville, NY). Images were captured using a Nikon Digital Sight DS-U2 camera, and analyzed using NIS element F.

### Pathology evaluation and immunohistochemistry

To validate the prognostic values of the selected candidate proteins, immunohistochemical studies were performed on tissue specimens from 113 patients who had been diagnosed with primary HG NMIBC following complete TURBT between 2000 and 2011 at Eulji University Hospital. The patients in the validation cohort underwent surveillance according to our follow-up protocol [9, 21]. Patients with short-term follow-up periods (less than 6 months) were excluded. All pathology slides prepared from each TURBT specimen were thoroughly reevaluated by a single uropathologist (JHK). Lymphovascular invasion (LVI) was defined as the observation of tumor cells in the luminal space lined by endothelial cells on hematoxylin and eosin staining [17].

Immunohistochemical staining and evaluation were performed as described previously [9, 21, 22]. Polyclonal rabbit anti-CREBBP (1:100) and anti-CD81 (1:100) antibodies were used. The optimal primary antibody dilutions were predetermined using appropriate positive controls (prostate and breast cancer tissues for CREBBP and CD81, respectively) and negative controls (omission of primary antibody). In our prior validation study of preanalytical factors [21], we confirmed high concordance rates for immunohistochemical staining (κ > 0.830, p < 0.001). Immunoreactivity was evaluated by light microscopy twice, at 4-week intervals, by a single uropathologist (JHK) who was blinded to clinical outcomes. A repeat reading of the same sample showed high concordance (κ = 0.872, p < 0.001). Immunoreactivity for each marker was evaluated semiquantitatively as previously described [21, 22]. Staining intensity was evaluated and reported on a scale of 0 to 3 (0, negative; 1, weak; 2, moderate; 3, strong); and according to the percentage of positively stained cells, samples were assigned a value of 1 (≤24%), 2 (25%–49%), 3 (50%–74%), or 4 (≥75%). An immunohistochemical score was calculated by multiplying the stained area by the intensity scores and was classified as negative (0–1), mild (2–3), moderate (4–8), or strong (9–12) expression.

### Statistical analysis

Outcomes were recurrence-free survival (RFS) and progression-free survival (PFS). Tumor recurrence was defined as the presence of pathological evidence of similar- or lower-stage disease by bladder biopsy or TURBT, and progression was defined as a pathological shift to more advanced-stage disease. Fisher’s exact test was used to evaluate the association between categorical variables. The Kaplan-Meier method was used to calculate RFS and PFS rates, and differences were evaluated with the log-rank test. For the analysis, biomarker expression based on immunohistochemical score was appropriately dichotomized because such groupings showed the most significant survival difference in the Kaplan-Meier analysis. The prognostic significance of candidate biomarkers and clinical parameters was assessed by univariate and multivariate Cox regression analysis. All tests were 2-sided, and p values less than 0.05 were considered significant. All statistical analyses were performed using Stata/SE software, version 12.1 (Stata Corporation, College Station, TX).

## Results

### Differential protein expression identified by AbM profiling

**Fig 1:**
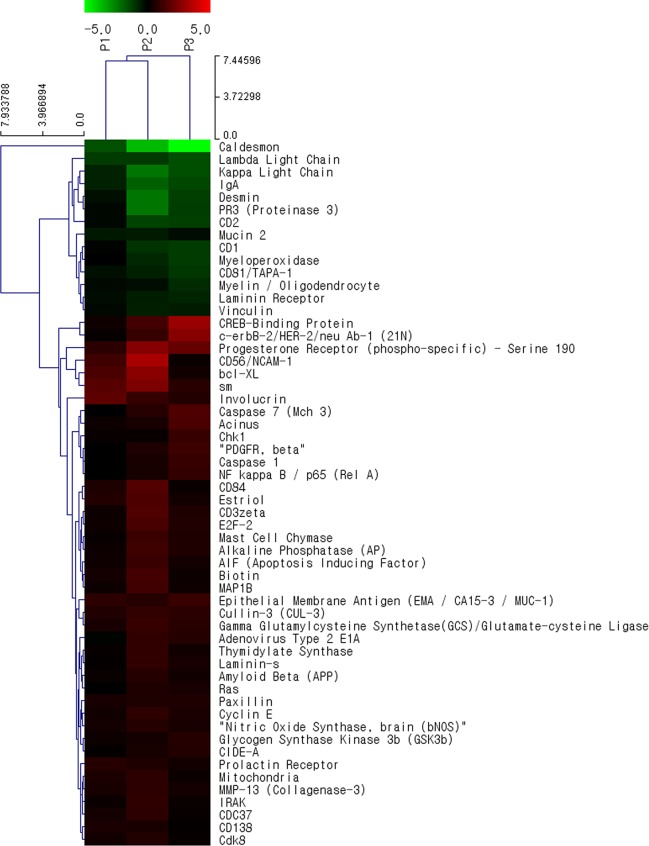
Differential protein expression between human bladder cancer (BC) tissues and normal urothelium identified by antibody microarray profiling. Three paired BC tissue and normal urothelium samples were obtained from 3 patients who were diagnosed with primary high grade non-muscle invasive BC following complete transurethral resection of the bladder tumor. Protein expression was analyzed by antibody microarray. Proteins shown in the right column are those with a >1.33 fold (or <0.77) change with p values <0.25. Red indicates higher expression in cancer tissues compared to normal bladder mucosa; green indicates lower expression in cancer tissues. Further details are available through GEO Series accession number GSE59440 (http://www.ncbi.nlm.nih.gov/geo/query/acc.cgi?acc=GSE59440).

Differentially expressed proteins between BC and normal urothelium were selected based on the fold change criteria (>1.33-fold or <0.77-fold). We found 42 upregulated proteins and 14 downregulated proteins with statistical significance in the BC tissues (Fig 1). While some proteins (shown in red in Fig 1) such as CREBBP, progesterone receptor, CD56, caspase 7, caspase 1, and PDGFR-β were more highly expressed in BC tissue than in normal mucosa, other proteins (shown in green in Fig 1), including caldesmon, desmin, myeloperoxidase, and CD81, had lower expression in BC tissue. Some proteins that appeared to not be associated with cancer development, such as IgA and light chain, were excluded as potential candidate biomarkers.

After an extensive, critical literature review focused on the association of the aforementioned differentially expressed proteins with cancer development and prognostic value in solid cancers, including BC [23–31], we identified CREBBP and CD81, which have been investigated in a limited number of BC studies (less than 3), as representative upregulated and downregulated candidate biomarkers, respectively.

### Evaluation of candidate protein expression in human cell lines

**Fig 2:**
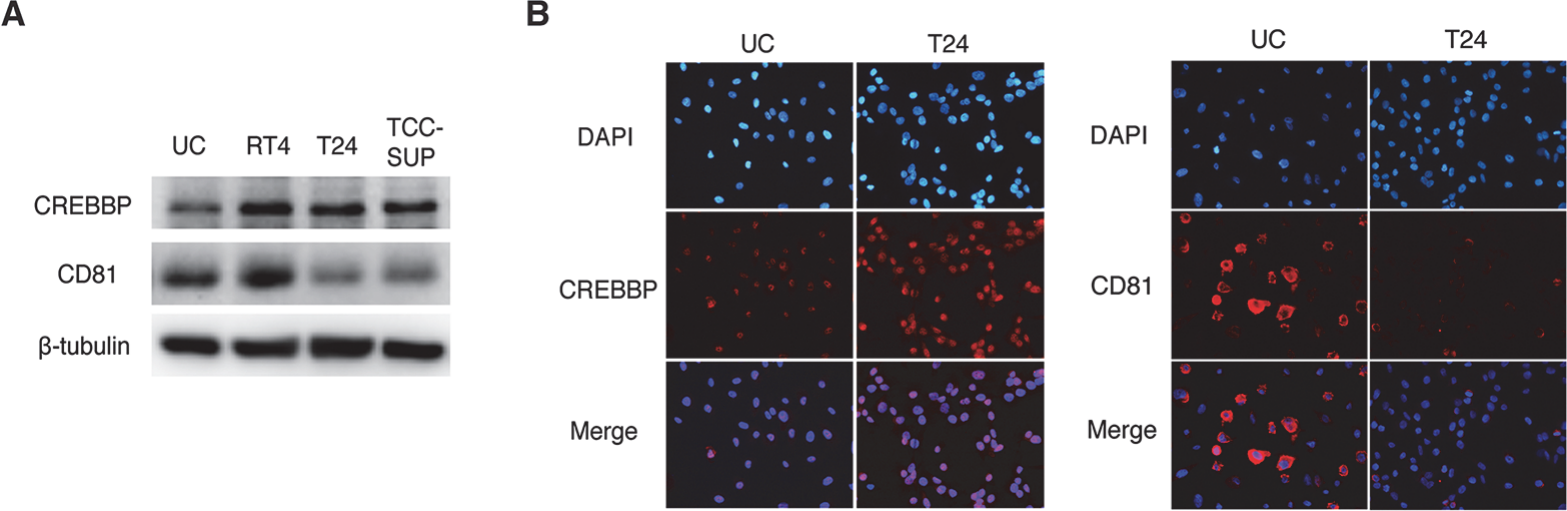
Protein expression of candidate biomarkers in human cell lines. (A) Total protein levels of CREB-binding protein (CREBBP) and CD81 were analyzed by western blotting. β-Tubulin was used as a calibration control. (B) The expression of CREBBP and CD81 were examined by immunofluorescence assay. CREBBP expression was mainly detected in the nucleus, and its expression was higher in T24 cells than in primary human urothelial cells. CD81 expression was prominent at the cell surface of primary human urothelial cells, but was only detected in a few T24 cells. Representative images from 3 independent experiments are shown. Nuclei and target proteins were detected using immunofluorescence staining and observed with a Nikon Eclipse E400 microscope at 400× magnification. UC, primary human urothelial cells.

To validate the expression of the candidate biomarkers in human cells, we performed western blotting and IFA (Fig 2A and 2B). The levels of CREBBP expression in 3 BC cell lines (RT4, T24, and TCC-SUP) were higher than those in primary human urothelial cells. The expression of CD81 in all tested BC cell lines, except the LG cell line RT4 [32, 33], were significantly lower than that in primary urothelial cells. Similar expression patterns were observed by IFA (Fig 2B). CREBBP expression was mainly detected in the nucleus, and its expression was higher in T24 cells than in primary human urothelial cells. CD81 expression was prominent at the cell surface of primary human urothelial cells, whereas it was only detected in a few T24 cells.

### Immunohistochemical staining for candidate biomarkers

**Fig 3:**
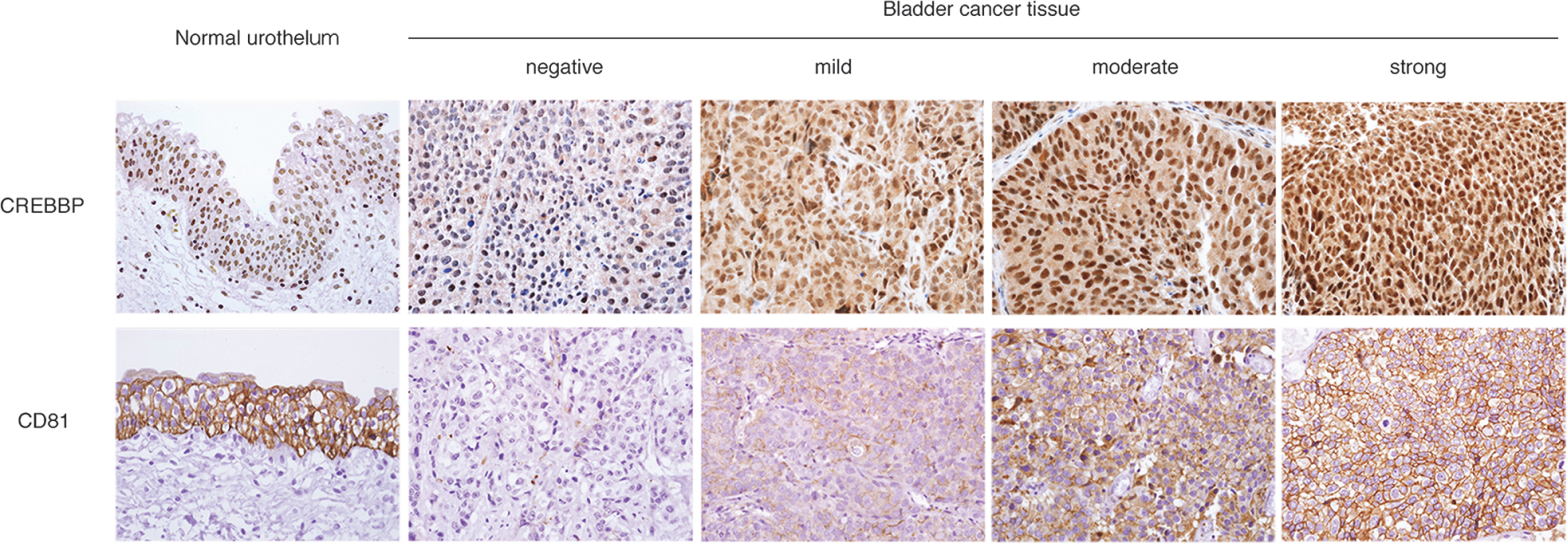
Immunohistochemical expression of candidate biomarkers in primary high grade non-muscle invasive bladder cancer (BC) tissues (magnification, 400×). CREB-binding protein (CREBBP) was expressed primarily in the nuclei of BC cells. CD81 was expressed primarily on the cytoplasmic membrane of normal urothelium, whereas its expression was much lower in BC tissues.

CREBBP and CD81 expression was also examined in human tissues by immunohistochemistry (Fig 3). CREBBP was expressed primarily in the nuclei of BC cells, whereas its expression was weak or absent in normal urothelium. CD81 was highly expressed on the cytoplasmic membrane of normal urothelium, whereas its expression was much lower in BC cells, although there was some variation in BC tissues. The immunohistochemical scores for the 2 biomarkers, based on staining area and intensity, were as follows: CREBBP expression was negative in 73 patients (64.6%), mild in 31 patients (27.4%), moderate in 5 patients (4.4%), and strong in 4 patients (3.5%); CD81 expression was negative in 8 patients (7.1%), mild in 21 patients (18.6%), moderate in 22 patients (19.5%), and strong in 62 patients (54.9%). Based on these data, CREBBP expression was dichotomized as negative or ≥mild (designated as “positive”), and CD81 expression was dichotomized as ≤moderate (designated as “low”) or strong (designated as “high”).

### Relationship between immunohistochemical biomarker expression and clinicopathological characteristics

To evaluate the relevance of CREBBP and CD81 as clinical biomarkers in BC, we analyzed expression in an independent HG NMIBC cohort by immunohistochemistry. Table 1 summarizes the baseline characteristics of the validation cohort. The median age of the patients was 68 years (range, 28–85 years). Positive CREBBP expression was significantly associated with adverse pathological characteristics, including multifocality, LVI, and higher T stage, whereas high CD81 expression was significantly associated with favorable pathological characteristics, including less carcinoma in situ, less LVI, papillary morphology, and lower T stage (Table 2). A significant inverse correlation was noted between CREBBP and CD81 expression (Kendall’s τ = -0.258, p = 0.006).

**Table 1:** Baseline characteristics of the validation cohort comprising 113 patients with primary high grade non-muscle invasive bladder cancer.

Variable	No. (%)
Gender	
Male	94 (83.2)
Female	19 (16.8)
Tumor size	
<3 cm	76 (67.3)
≥3 cm	37 (32.7)
Multifocality	
Single	77 (68.1)
Multiple	36 (31.9)
Concomitant carcinoma in situ	
No	91 (80.5)
Yes	22 (19.5)
Morphology	
Papillary	94 (83.2)
Sessile	19 (16.8)
Lymphovascular invasion	
No	86 (76.1)
Yes	27 (23.9)
Intravesical therapy	
No	50 (44.2)
Yes^*^	63 (55.8)
Tumor stage	
Ta	36 (31.9)
T1	77 (68.1)
Recurrence	
No	68 (60.2)
Yes	45 (39.8)
Progression	
No	93 (82.3)
Yes	20 (17.7)

^*^Among patients undergoing intravesical therapy, 59 (93.7%) underwent bacillus Calmette-Guérin instillations.

**Table 2:** Associations between biomarker expression and clinicopathological characteristics.

Variable	Biomarker expression^*^						
	CREBBP expression				CD81 expression		
	Negative	Positive	p		Low	High	p
Total no. (%)	73 (64.6)	40 (35.4)	-		51 (45.1)	62 (54.9)	-
Gender (no. [%])			0.503				0.771
Male	62 (84.9)	32 (80.0)			43 (84.3)	51 (82.3)	
Female	11 (15.1)	8 (20.0)			8 (15.7)	11 (17.7)	
Tumor size (no. [%])			0.967				0.600
<3 cm	49 (67.1)	27 (67.5)			33 (67.4)	43 (69.4)	
≥3 cm	24 (32.9)	13 (32.5)			18 (35.3)	19 (30.6)	
Multifocality (no. [%])			0.008				0.128
Single	56 (76.7)	21 (52.5)			31 (60.8)	46 (74.2)	
Multiple	17 (23.3)	19 (47.5)			20 (39.2)	16 (25.8)	
Concomitant carcinoma in situ (no. [%])			0.272				0.004
No	61 (83.6)	30 (75.0)			35 (68.6)	56 (90.3)	
Yes	12 (16.4)	10 (25.0)			16 (31.4)	6 (9.7)	
Morphology (no. [%])			0.885				0.025
Papillary	61 (83.6)	33 (82.5)			38 (74.5)	56 (90.3)	
Sessile	12 (16.4)	7 (17.5)			13 (25.5)	6 (9.7)	
Lymphovascular invasion (no. [%])			0.003				<0.001
No	62 (84.9)	24 (60.0)			28 (54.9)	58 (93.5)	
Yes	11 (15.1)	16 (40.0)			23 (45.1)	4 (6.5)	
Intravesical therapy (no. [%])			0.063				0.829
No	37 (50.7)	13 (32.5)			22 (43.1)	28 (45.2)	
Yes	36 (49.3)	27 (67.5)			29 (56.9)	34 (54.8)	
Stage (no. [%])			0.001				<0.001
Ta	31 (42.5)	5 (12.5)			3 (5.9)	33 (53.2)	
T1	42 (57.5)	35 (87.5)			48 (94.1)	29 (46.8)	

Abbreviations: CREBBP, CREB-binding protein

^*^The immunohistochemical score was based on staining area and intensity, and expression was dichotomized based on the groupings that showed the most significant survival difference in the Kaplan-Meier analysis.

### Prognostic value of the candidate biomarkers

The median follow-up was 48.0 months (mean, 49.7 months; range, 6–138.6 months). During surveillance, recurrence and progression were observed in 45 (39.8%) and 20 (17.7%) patients, respectively at median times of 14.0 and 32.9 months, respectively. The overall 5-year RFS and PFS rates were 65.6% and 82.1%, respectively.

**Fig 4:**
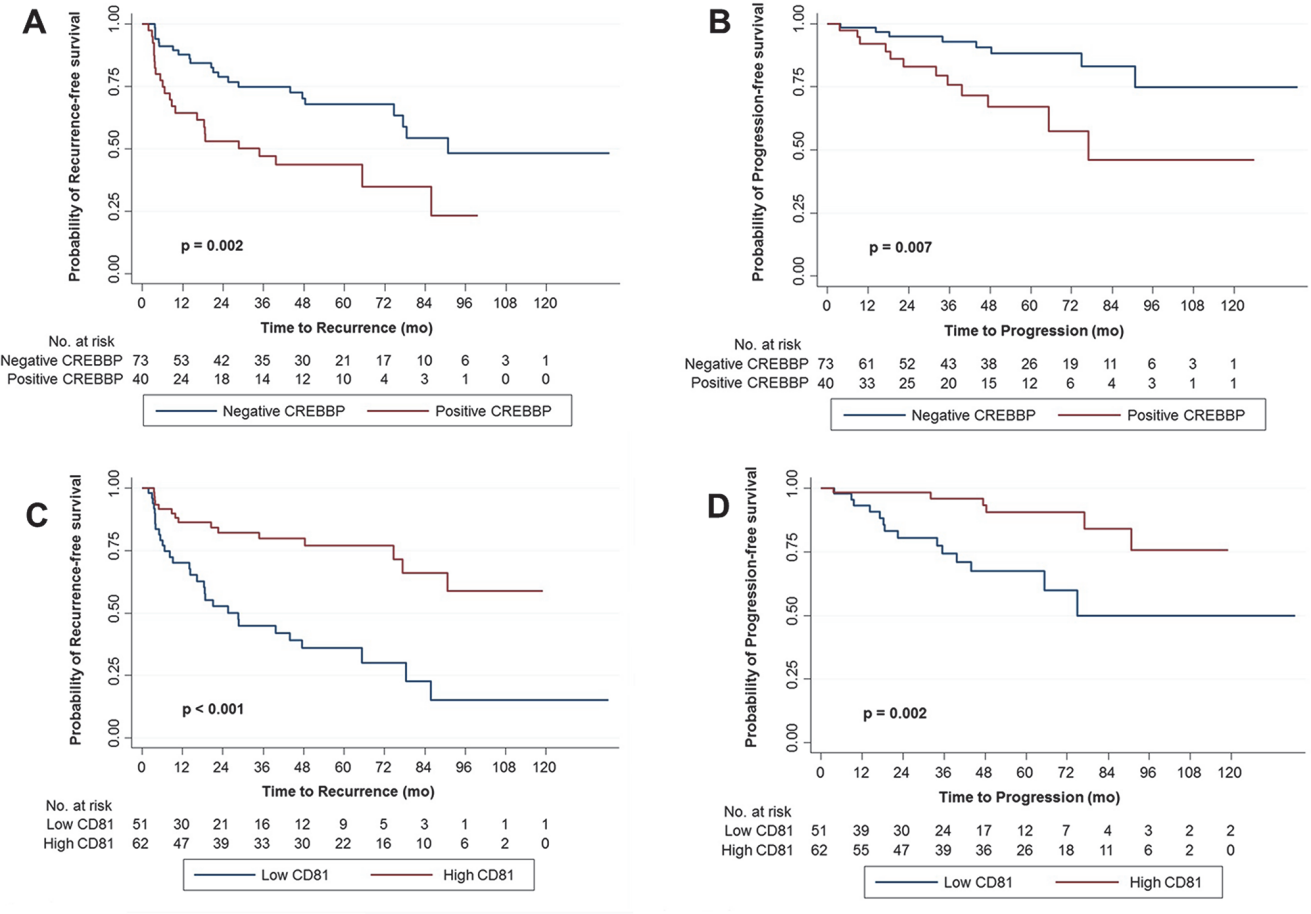
Kaplan-Meier survival curves for recurrence-free survival (RFS) and progression-free survival (PFS) according to CREBBP and CD81 expression. Expression was dichotomized according to the immunohistochemical score, which was based on staining area and intensity. (A) RFS and (B) PFS according to CREBBP expression, (C) RFS and (D) PFS according to CD81 expression.

In the Kaplan-Meier survival analysis, positive CREBBP expression was significantly associated with lower RFS and PFS rates than negative expression (Fig 4A and 4B), whereas high CD81 expression was significantly associated with higher RFS and PFS rates than low expression (Fig 4C and 4D). Similarly, univariate Cox regression analyses showed that CREBBP and CD81 expression were significant predictors for tumor recurrence and progression (Table 3). Compared to negative expression, positive CREBBP expression was associated with a 2.417-fold increased risk for tumor recurrence (p = 0.003) and a 3.213-fold increased risk for progression (p = 0.011). Conversely, compared to low CD81 expression, high CD81 expression was associated with a 0.650-fold decreased risk for tumor recurrence (p < 0.001) and a 0.630-fold decreased risk for progression (p = 0.005).

**Table 3:** Univariate and multivariate Cox regression analyses of multiple variables for recurrence and progression-free survival.

Variable	Recurrence-free survival				Progression-free survival		
	HR	95% CI	p		HR	95% CI	p
**Univariate analysis**							
Clinicopathological parameters							
Age (as continuous variable)	1.022	0.997–1.048	0.084		1.041	0.999–1.085	0.055
Gender (male vs female)	1.097	0.528–2.283	0.803		2.893	1.177–7.110	0.021
Tumor size (<3 cm vs. ≥3 cm)	2.241	1.234–4.070	0.008		1.760	0.715–4.329	0.219
Multifocality (single vs. multiple)	2.083	1.149–3.774	0.016		2.284	0.941–5.534	0.068
Concomitant carcinoma in situ (no vs. yes)	1.435	0.709–2.904	0.315		3.959	1.599–9.801	0.003
Morphology (papillary vs. non-papillary)	1.685	0.798–3.556	0.171		3.688	1.332–10.212	0.012
Lymphovascular invasion (no vs. yes)	1.675	0.877–3.198	0.118		2.250	0.889–5.698	0.087
Intravesical therapy (no vs. yes)	0.586	0.325–1.055	0.075		0.518	0.212–1.270	0.151
T stage (Ta vs. T1)	2.591	1.229–5.463	0.012		3.946	1.136–13.708	0.031
Biomarkers^*^							
CREBBP (negative vs. positive expression)	2.417	1.341–4.356	0.003		3.213	1.307–7.899	0.011
CD81 (low vs. high expression)	0.650	0.528–0.801	<0.001		0.630	0.457–0.868	0.005
**Multivariate analysis**							
Gender (male vs. female)	1.027	0.446–2.366	0.950		2.636	0.867–8.018	0.088
Tumor size (<3 cm vs. ≥3 cm)	2.279	1.150–4.518	0.018		1.788	0.681–4.696	0.238
Multifocality (single vs. multiple)	2.598	1.204–5.607	0.015		2.691	0.888–8.151	0.080
Concomitant carcinoma in situ (no vs. yes)	1.194	0.461–3.093	0.715		2.832	0.855–9.384	0.088
Morphology (papillary vs. non-papillary)	1.162	0.436–3.099	0.764		1.458	0.379–5.610	0.583
Intravesical therapy (no vs. yes)	0.252	0.116–0.548	<0.001		0.275	0.087–0.875	0.029
T stage (Ta vs. T1)	0.942	0.340–2.608	0.908		1.706	0.341–8.539	0.516
CREBBP (negative vs. positive expression)	2.029	1.005–4.097	0.048		1.714	0.537–5.473	0.363
CD81 (low vs. high expression)	0.727	0.558–0.946	0.018		0.838	0.563–1.247	0.363

Abbreviations: CREBBP, CREB-binding protein; HR, hazard ratio; CI, confidence interval.

^*^The immunohistochemical score was based on staining area and intensity, and expression was dichotomized based on the groupings that showed the most significant survival difference in the Kaplan-Meier analysis.

**Fig 5:**
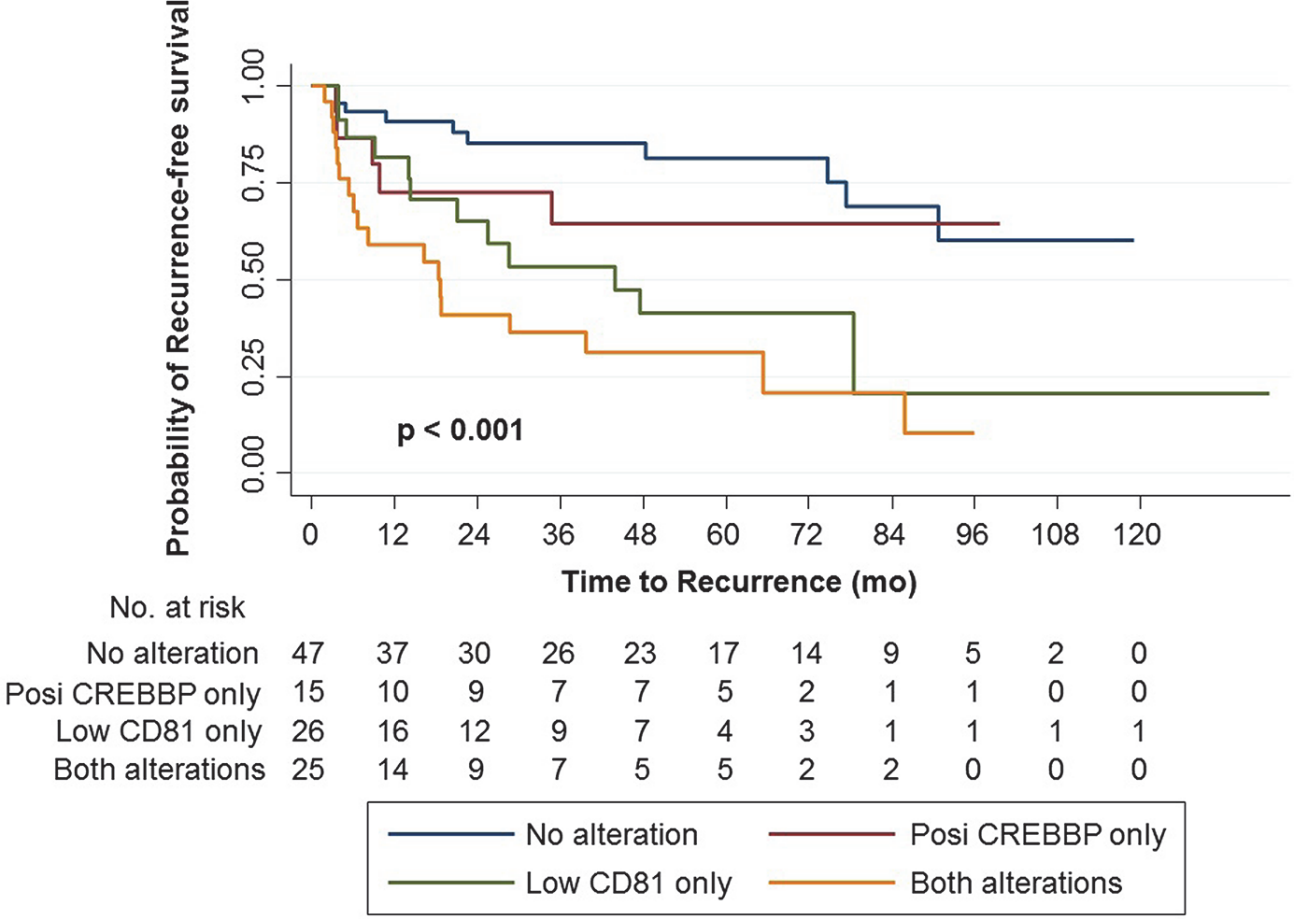
Kaplan-Meier survival curves for recurrence-free survival (RFS) according to the number of biomarkers showing altered expression (i.e., positive expression of CREBBP or low expression of CD81).

In a multivariate analysis including both biomarkers, the 2 biomarkers remained significant predictors for RFS. CREBBP and CD81 alterations were associated with a 2.029-fold (p = 0.048) and 0.727-fold (p = 0.018) increased risk for tumor recurrence, respectively (Table 3). In multivariate analysis with each biomarker alone, similar findings were obtained (S1 Table). In multivariate analysis for PFS, intravesical therapy only was significant for PFS (HR = 0.275, p = 0.029), and the 2 biomarkers were not significant (Table 3). Survival analysis according to the 2 prognostic biomarkers indicated that RFS significantly decreased with an increased number of marker alterations (Fig 5).

## Discussion

This study showed, for the first time, the use of an AbM to identify novel candidate biomarkers that can differentiate between BC and normal urothelium. The 2 candidate proteins chosen from the AbM analysis, CREBBP and CD81, were validated in BC cell lines and human tissue samples. In addition, we confirmed the prognostic significance of the candidate proteins in primary HG NMIBC. These findings indicate potential applicability of AbM profiling for the identification of novel biomarkers in primary HG NMIBC.

AbM profiling has the advantage that it directly measures protein expression. Although cDNA microarrays are commonly used to evaluate mRNA expression, gene expression profiling may not represent the cellular proteome because posttranslational modifications cannot be detected. We found that the protein expression from the AbM analysis was relatively reliable correlated with the results of western blotting/IFA of cell lines and immunohistochemistry on BC tissue samples. However, the expression of the candidate proteins identified by the AbM profiling still needed to be confirmed in additional experiments. Because AbMs are based on protein extracts, the microarrays do not provide information about cell type (e.g., epithelial or stroma cells) or the location (e.g., cytosol or nucleus) of the protein. For example, PDGFR-β expression, which was recently suggested as a prognostic biomarker in NMIBC [31], was not detected in BC tissues or in normal urothelial tissues and cells even though this protein was upregulated in our AbM profiling (Fig 1). Interestingly, immunohistochemical staining showed that PDGFR-β was only located in BC stromal tissues (S1 Fig).

CREBBP is highly homologous and functionally related to p300, which was originally identified as a protein that interacts with the adenoviral E1A oncoprotein [34, 35]. CREBBP is involved in multiple signal transduction pathways where is functions as a transcription cofactor for genes that encode many different proteins, such as oncoproteins, transforming viral proteins, and tumor-suppressor proteins. CREBBP has been implicated in the carcinogenesis of several cancers [23–25]. For example, genomic alteration of CREBBP is associated with esophageal cancer [24] and small cell lung cancer [25]. CREBBP expression has also been shown to be associated with prognosis of small cell lung cancer [25]. Positive CREBBP expression was associated with a significantly lower survival than negative CREBBP expression. To date, only one study has examined the expression of CREBBP in human BC cell lines and radical cystectomy tissue specimens [26]. The study [26] included both NMIBC (40% of the patient population) and muscle-invasive BC (stage T2 or higher; 60% of the patient population), positive CREBBP expression was noted in 86% of patients (47/55), which was significantly higher than that in ours (35.4%). Although differences in patient populations preclude direct comparison between the two studies [26], these findings suggest that MIBC has a higher proportion of CREBBP positivity than NMIBC. No previous study has examined the prognostic value of CREBBP expression in BC patients. Our results suggest that positive CREBBP expression is significantly associated with adverse pathological characteristics and poor prognosis in primary HG NMIBC. Since CREBBP is a transcription cofactor for many different proteins, further studies are needed to elucidate the underlying mechanism in BC.

CD81, a member of the tetraspanin family, is a cell-surface protein that is involved in a broad range of cellular functions, including development, activation, growth, and motility. With regard to cancer pathogenesis, in several tumors, CD81 has been shown to play a role in cancer cell migration and progression; in particular, decreased expression of CD81 is associated with metastasis in hepatocellular carcinoma and with the growth and survival of gastric cancer [27, 28]. In a recent study, a direct interaction between the C-terminal cytoplasmic domain of CD81 and Rac has been suggested as a possible mechanism for regulating cell migration [29]. Studies on the role of CD81 in BC carcinogenesis and its prognostic value are sparse; however, one study reported heterogeneous CD81 and CD82 expression in human BC cell lines [30], but its clinical significance was not investigated. To our knowledge, this study is the first to demonstrate an inverse correlation between CD81 expression and adverse pathological characteristics and its prognostic value in NMIBC. Further studies are required to reveal the exact mechanisms underlying the role of CD81 in BC carcinogenesis and prognosis.

Our validation cohort results clearly show that CREBBP and CD81 have prognostic value for predicting the clinical outcomes of 113 patients with primary HG NMIBC. Significantly, our RFS analysis with both prognostic biomarkers (Fig 5) suggested that the 2 biomarkers may have a cumulative effect on patient prognosis, even though the markers are involved in different cellular processes. The lack of statistical significance in the PFS analysis was likely due, at least in part, to the relatively small number of progression events in our validation cohort.

In summary, this study demonstrated the potential applicability of AbM profiling for identifying novel biomarkers in NMIBC. Alterations in the expression of 2 biomarkers, CREBBP and CD81, identified in our study were associated with biologic aggressiveness of tumors and poor prognosis in primary HG NMIBC. Although further external validations are required in larger cohorts, we suggest that evaluation of the expression of CREBBP and CD81 in TURBT specimens has useful prognostic value in patients with primary HG NMIBC and that patients with positive CREBBP and low CD81 expression may require closer surveillance and more aggressive treatment.

## Supporting Information

S1 Table Multivariate Cox regression analyses with each biomarker added for recurrence and progression-free survival (DOCX)

S1 Fig Immunohistochemical expression of PDGFR-β in bladder cancer tissue. (A) 100× Magnification. (B) 200× Magnification. PDGFR-β immunoreactivity was observed in stromal tissues (arrows) but not in epithelial cells.
(TIF)
