# Supplementary material for: Prognostic Significance of CREB-Binding Protein and CD81 Expression in Primary High Grade Non-Muscle Invasive Bladder Cancer: Identification of Novel Biomarkers for Bladder Cancer Using Antibody Microarray
Source: PLoS One. 2015 Apr 27;10(4):e0125405. doi: 10.1371/journal.pone.0125405 (PMC4411067; doi:10.1371/journal.pone.0125405)
Supplement: S1 Table — (DOCX) [file pone.0125405.s001.docx]

Table S1. Multivariate Cox regression analyses, with each biomarker added for recurrence and progression-free survival

| Variables | Recurrence-free survival | | |  | Progression-free survival | | |
| --- | --- | --- | --- | --- | --- | --- | --- |
|  | HR | 95% CI | p |  | HR | 95% CI | p |
| **Multivariate analysis (CREBBP added)** | | | | | | | |
| Gender (male vs. female) | 1.039 | 0.456–2.367 | 0.927 |  | 2.786 | 0.935–8.304 | 0.066 |
| Tumor size (<3 cm vs. ≥3 cm) | 2.189 | 1.109–4.321 | 0.024 |  | 1.758 | 0.667–4.636 | 0.254 |
| Multifocality (single vs. multiple) | 2.537 | 1.223–5.262 | 0.012 |  | 2.609 | 0.886–7.684 | 0.082 |
| Concomitant carcinoma in situ (no vs. yes) | 1.210 | 0.467–3.132 | 0.695 |  | 2.934 | 0.885–9.724 | 0.078 |
| Morphology (papillary vs. nonpapillary) | 1.479 | 0.559–3.913 | 0.431 |  | 1.648 | 0.434–6.265 | 0.463 |
| Intravesical therapy (no vs. yes) | 0.256 | 0.125–0.526 | <0.001 |  | 0.284 | 0.095–0.849 | 0.024 |
| T stage (Ta vs. T1) | 1.646 | 0.697–3.888 | 0.256 |  | 2.152 | 0.489–9.476 | 0.311 |
| CREBBP^*^ (negative vs. positive expression) | 2.318 | 1.154–4.656 | 0.018 |  | 1.930 | 0.625–5.959 | 0.253 |
| **Multivariate analysis (CD81 added)** | | | | | | | |
| Gender (male vs. female) | 1.210 | 0.533–2.750 | 0.649 |  | 3.129 | 1.093–8.961 | 0.034 |
| Tumor size (<3 cm vs. ≥3 cm) | 2.179 | 1.102–4.308 | 0.025 |  | 1.788 | 0.680–4.700 | 0.238 |
| Multifocality (single vs. multiple) | 3.177 | 1.467–6.880 | 0.003 |  | 3.133 | 1.059–9.274 | 0.039 |
| Concomitant carcinoma in situ (no vs. yes) | 1.216 | 0.465–3.180 | 0.690 |  | 2.927 | 0.871–9.838 | 0.083 |
| Morphology (papillary vs. nonpapillary) | 1.091 | 0.409–2.913 | 0.861 |  | 1.356 | 0.350–5.251 | 0.659 |
| Intravesical therapy (no vs. yes) | 0.266 | 0.121–0.582 | 0.001 |  | 0.272 | 0.085–0.871 | 0.028 |
| T stage (Ta vs. T1) | 1.210 | 0.467–3.135 | 0.695 |  | 2.188 | 0.493–9.716 | 0.303 |
| CD81^*^ (low vs. high expression) | 0.709 | 0.550–0.913 | 0.008 |  | 0.807 | 0.551–1.181 | 0.269 |

Abbreviations: CREBBP, CREB-binding protein; HR, hazard ratio; CI, confidence interval.

^*^The immunohistochemical score was based on staining area and intensity, and expression was dichotomized accordingly because such grouping showed the most significant survival difference on the Kaplan–Meier analysis.
