# Supplementary figures and images for: Prognostic Significance of CREB-Binding Protein and CD81 Expression in Primary High Grade Non-Muscle Invasive Bladder Cancer: Identification of Novel Biomarkers for Bladder Cancer Using Antibody Microarray
Source: PLoS One. 2015 Apr 27;10(4):e0125405. doi: 10.1371/journal.pone.0125405 (PMC4411067; doi:10.1371/journal.pone.0125405)

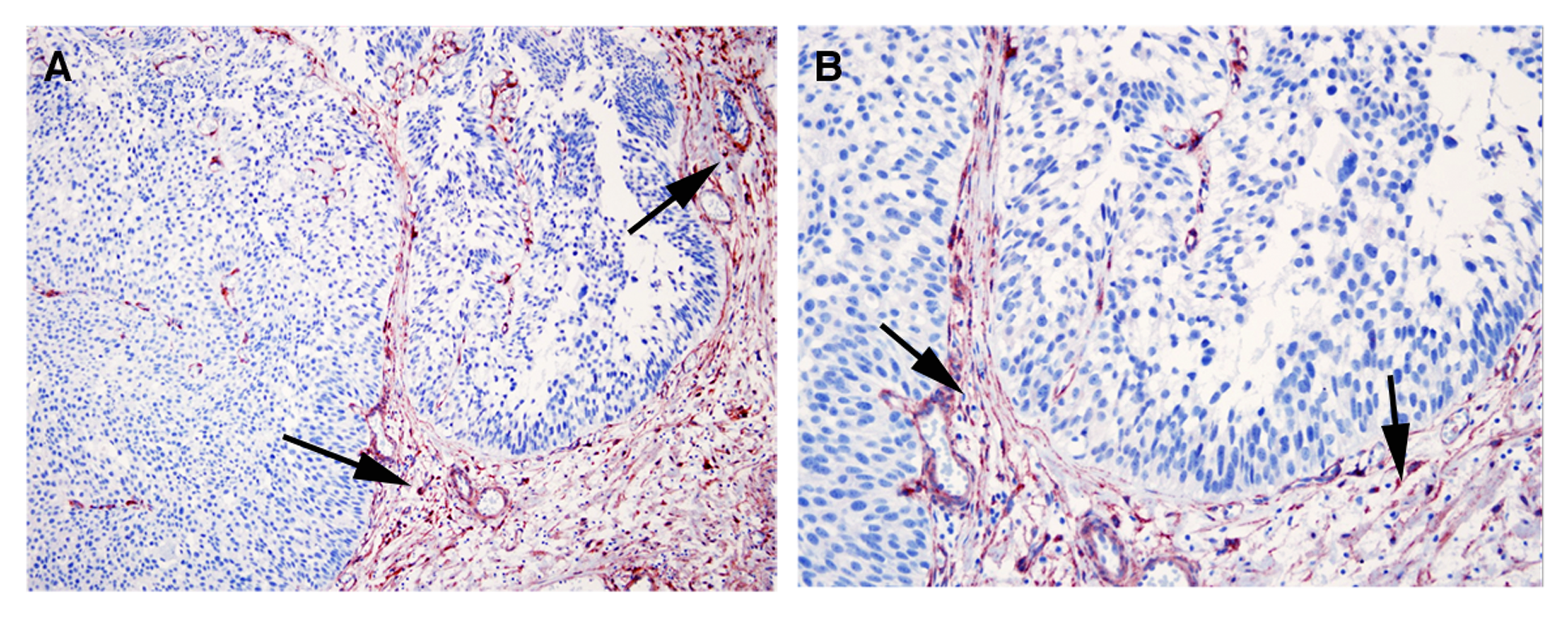

Supplement: S1 Fig — (A) 100× Magnification. (B) 200× Magnification. PDGFR-β immunoreactivity was observed in stromal tissues (arrows) but not in epithelial cells. (TIF) [file pone.0125405.s002.tif]
